# A Single Coronary Artery Anomaly: Right Coronary Artery as a Branch From the Left Anterior Descending Artery

**DOI:** 10.7759/cureus.9801

**Published:** 2020-08-17

**Authors:** Mohammed Salih, Osama Abdel-Hafez, Ramzi Ibrahim, Abdul R Halabi, Feras Aloka

**Affiliations:** 1 Internal Medicine, Saint Joseph Mercy Oakland, Pontiac, USA; 2 Cardiology, Saint Joseph Mercy Oakland, Pontiac, USA

**Keywords:** cardiology

## Abstract

An anomalous origin of the right coronary artery (RCA) from the left anterior descending artery (LAD), also known as a single coronary artery, is an extremely rare finding in clinical practice. It is usually a benign anomaly; however, symptoms are highly dependent on the course that the anomalous RCA takes after branching off of the LAD. We present a case of a patient who had decompensated heart failure and was detected to have a single coronary artery. The patient was treated with guideline-directed medical therapy with notable improvement in clinical status in the following days. Enhanced awareness of congenital cardiac anomalies may help guide management.

## Introduction

Congenital coronary anomalies include a wide range of phenotypic findings. It can be asymptomatic; however, some patients present with angina, syncope, or sudden cardiac death. They are usually found incidentally on cardiac angiography or coronary CT. The reported incidence of coronary arteries anomalies is 1.3% [1,2]. The incidence of an anomalous origin of the right coronary artery (RCA) from the left anterior descending artery (LAD) is 0.26% [3]. This anomaly is an extremely rare finding, and is also typically benign in nature, though highly dependent on the anatomical course. This congenital phenomenon is also known as a single coronary artery. In our case, we describe a patient who presented with symptomatic heart failure and was found to have a single coronary artery.

## Case presentation

A 62-year-old female patient with a medical history of chronic obstructive pulmonary disease (COPD) presented to the hospital with cough, dyspnea on exertion, and bilateral lower extremity swelling. Physical examination revealed vital signs significant for tachypnea with a respiratory rate of 24 breath/min, otherwise normal. Her general exam was significant for an elderly ill-looking female who was in acute distress with generalized edema. Her cardiopulmonary exam was significant for an elevated jugular venous pressure, regular rate and rhythm, normal first (S1) and second heart sounds (S2), and a third heart sound (S3) gallop was detected without a murmur. Crackles and reduced air entry at lung bases were appreciated. There was evident bilateral lower extremity edema noted with palpable peripheral pulses. The examination of the other systems was otherwise normal.　

**Figure 1:**
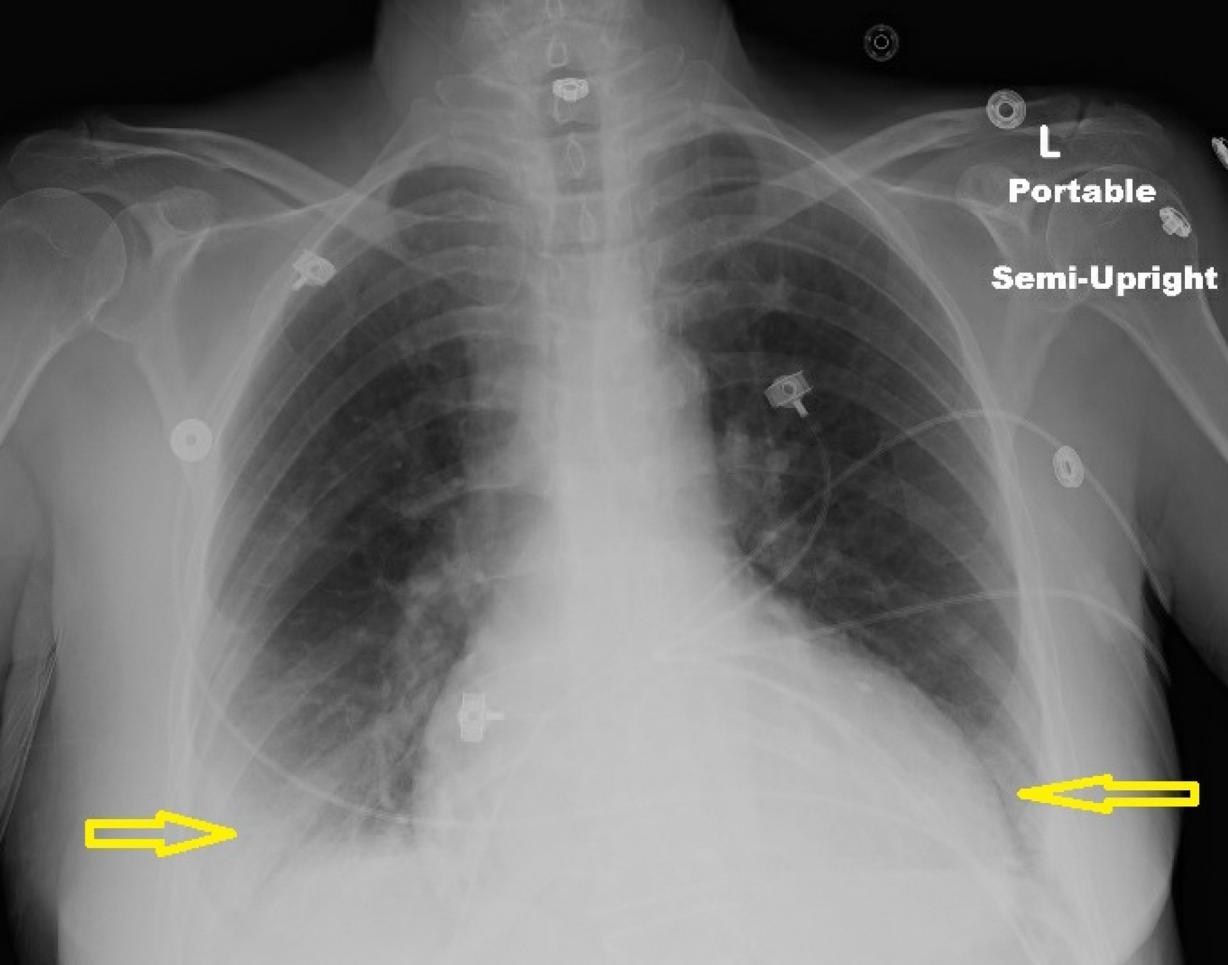
Chest x-ray showed bilateral pleural effusion greater on the right side with evidence of pulmonary vascular congestion.

Lab work was significant for a white blood cell count of 6,000/µL, platelets of 129,000/µL, and hemoglobin of 13.5 g/dL. Her metabolic panel was significant for sodium level of 133 mg/dL, chloride level of 98 mg/dL, and a normal albumin level at 3.9 g/dL. The rest of her metabolic panel was within normal limits. Her thyroid function tests were unremarkable. Brain natriuretic peptide was at 970 ng/L (normal <100 ng/L); troponin was noted to be 0.10 ng/mL (normal <0.03 ng/mL). She had negative tests for influenza A and B, Legionella, and Mycoplasma antigen. A urine drug screen was also negative. A chest x-ray showed bilateral pleural effusion greater on the right side with evidence of pulmonary vascular congestion (Figure 1). Echocardiogram showed an ejection fraction of 25% with moderate global left ventricular hypokinesia, grade 2 diastolic dysfunction, mild pulmonary valvular regurgitation with an otherwise normal aortic, mitral, and tricuspid valves. The patient was admitted for a diagnosis of acute decompensated cardiomyopathy.

**Figure 2:**
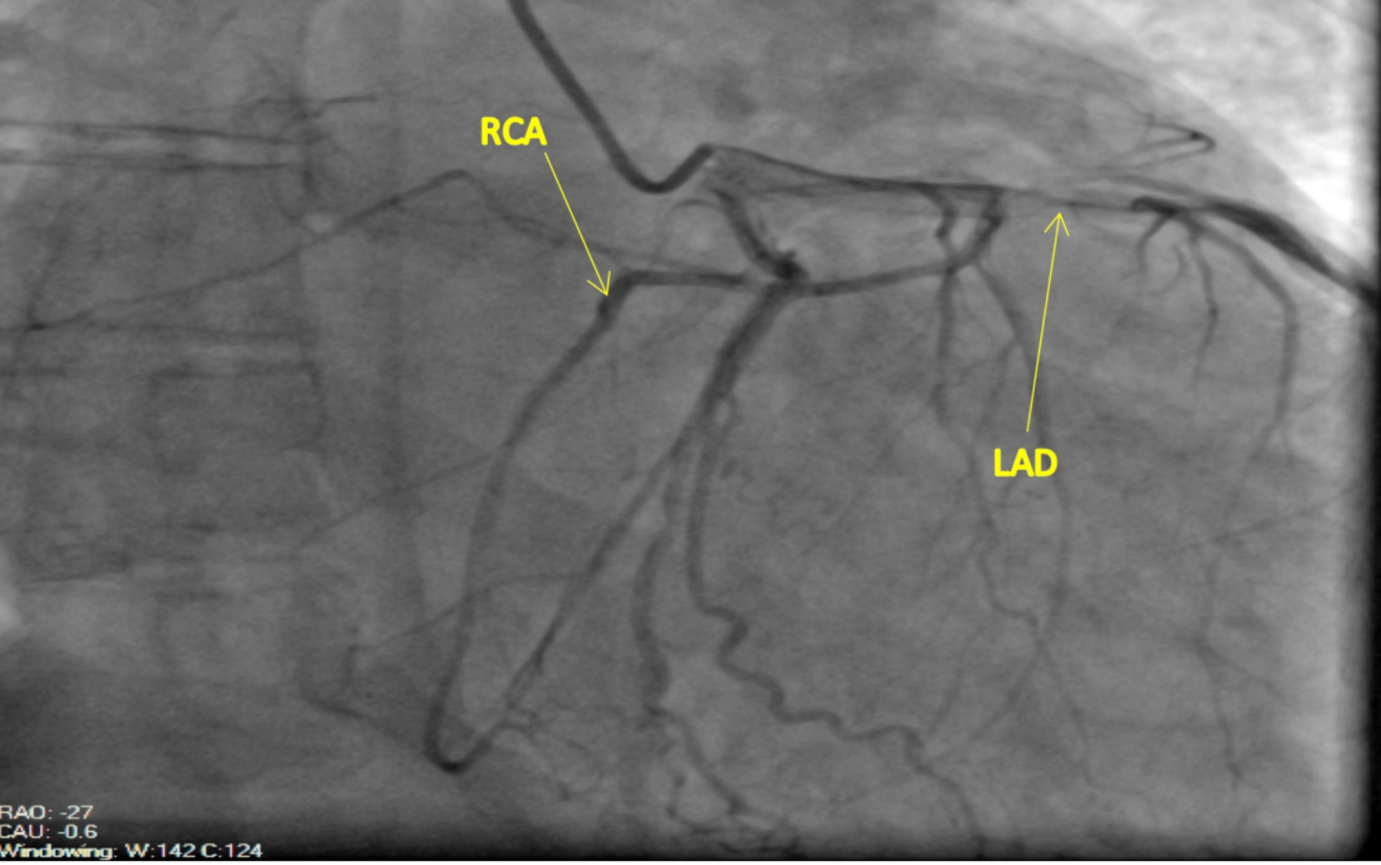
Left anterior oblique (LAO) projection: Anomalous origin of the RCA from the mid LAD. The anomalous RCA courses anteriorly across the right ventricular outflow tract in an epicardial fashion into the atrioventricular groove. RCA, right coronary artery; LAD, left anterior descending artery

**Figure 3:**
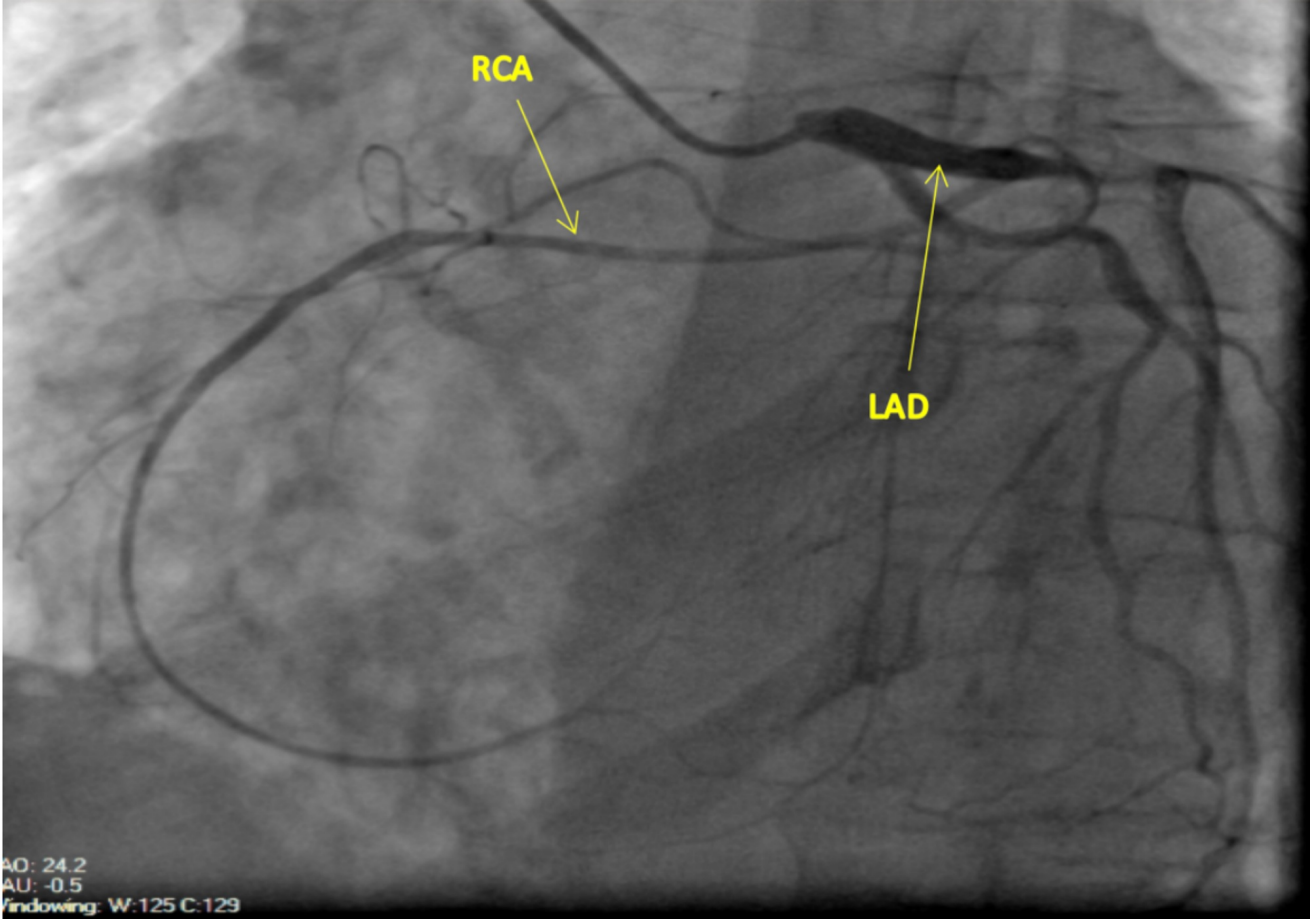
Right anterior oblique (RAO) projection: Anomalous origin of the RCA from the mid LAD. The anomalous RCA courses anteriorly across the right ventricular outflow tract in an epicardial fashion into the atrioventricular groove. RCA, right coronary artery; LAD, left anterior descending artery

**Figure 4:**
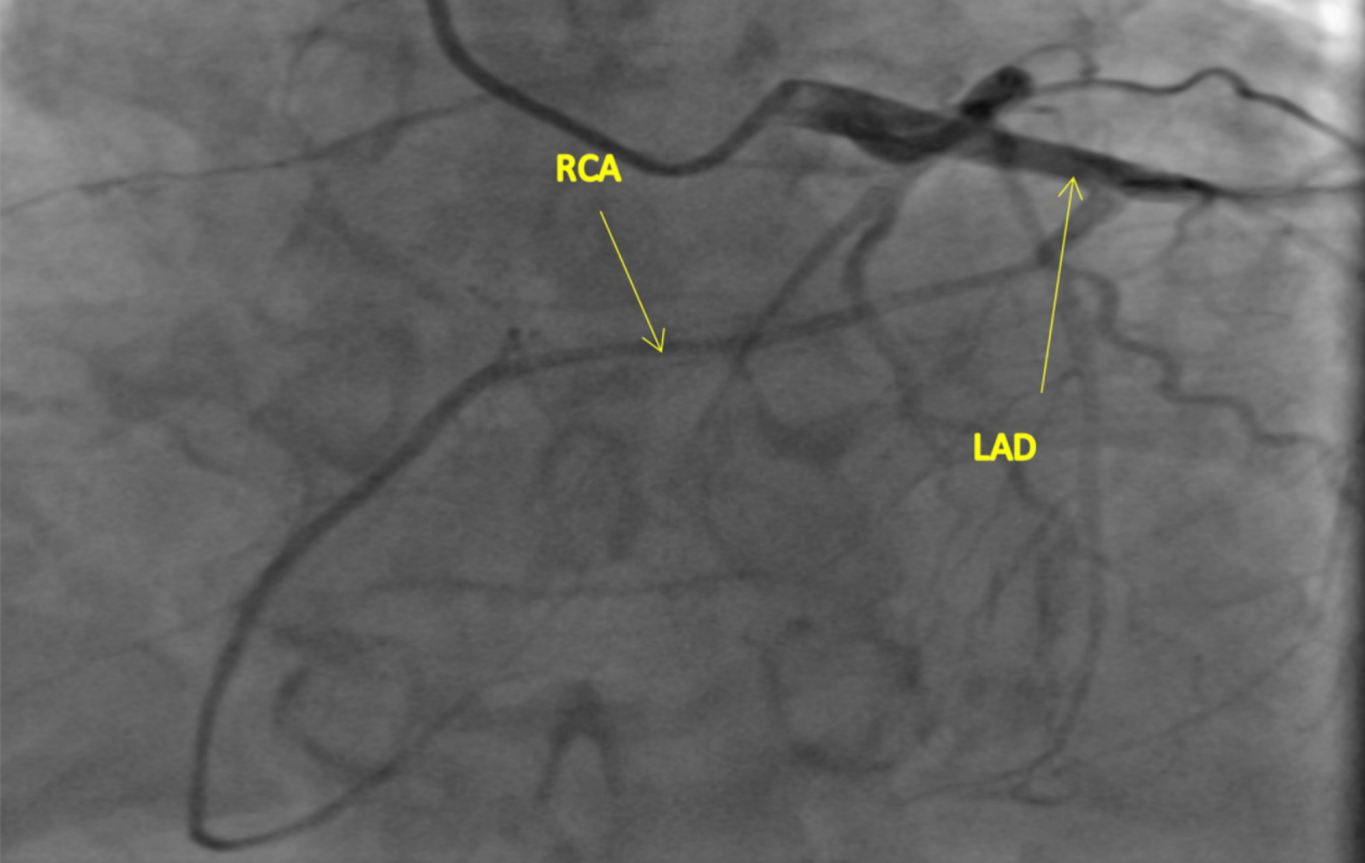
Anterior-posterior (AP) cranial projection: Anomalous origin of the RCA from the mid LAD. The anomalous RCA courses anteriorly across the right ventricular outflow tract in an epicardial fashion into the atrioventricular groove. RCA, right coronary artery; LAD, left anterior descending artery

Based on echocardiographic findings, cardiac catheterization was done for the evaluation of an ischemic cause of cardiomyopathy. The findings on the left heart catheterization revealed a patent left main coronary artery, which bifurcated into the left circumflex artery (LCX) and LAD. The LAD was patent proximally and gave rise to a medium-size diagonal one and diagonal two arteries, which were both patent. The mid to distal segment of the LAD and the LCX were also patent. The RCA was detected to have an anomalous origin from the mid LAD (Figures 2-4). The RCA showed evidence of mild diffuse disease in the proximal and mid segments. It also gave rise to a small posterior descending artery and first posterolateral branch. Based on the above findings, the diagnosis of non-ischemic cardiomyopathy (NICMP) was made, and the patient was informed about the diagnosis and the finding of the single coronary artery.

The patient was admitted and treated for acute systolic heart failure with guideline-directed medical therapy (GDMT), intravenous diuresis, and salt restriction. The patient showed significant clinical improvement over the next few days. The patient reported less dyspnea and improved functional status. There was also a significant decrease in the severity of the edema. She was successfully discharged on guideline-directed medical therapy and an oral diuretic regimen with plans for outpatient follow-up.

On the follow-up visit, the patient reported complete resolution of the dyspnea and edema. With a thorough clinical exam, she appeared to be compensated. GDMT was titrated as necessary and the patient was advised to follow up again in two months with plans to obtain coronary CT and repeat echocardiogram. She was also referred to a cardiac rehabilitation program.

## Discussion

Coronary anomalies are rare findings in clinical practice with an estimated incidence of 1.3% [1,2]. The most commonly reported coronary anomaly is an anomalous LCX arising from the RCA or the right coronary sinus [3,4]. Furthermore, the most common RCA anomaly is an RCA originating from the left main coronary artery [5,6]. Most coronary vessel anomalies are asymptomatic and found incidentally upon cardiac angiography. Although most are benign in nature with no underlying pathological consequences, serious outcomes may occur at a relatively significant rate of 19.4% of all patients with coronary anomalies [1].

In our patient, the RCA originates from the LAD. This is an extremely rare presentation with less than 50 cases reported in the medical literature [7]. This anomaly falls under the spectrum of a single coronary artery anomaly, indicating that the coronary arteries originate from a single coronary ostium within the aorta [8]. In cases where the RCA arises from the LAD, it usually arises after the first septal perforator branch [9,10]. Less commonly, it may arise from the proximal segment [10]. Moreover, this anomaly is most commonly associated with no other structural abnormalities or congenital heart disease [11].

The gold standard for diagnosing coronary artery anomalies is coronary angiography. The modified Lipton classification is the best available tool for the classification of single coronary artery anomalies. Each anomaly is denoted with an R or L based on the location of the sinus from which the coronary artery originates. Furthermore, the anomalies are classified into three subtypes; in type 1 anomalies the vessel follows the course of the normal left or right coronary artery. In type 2 anomalies, the anomalous vessel originates from the proximal part of the normal coronary artery. In type 3 anomalies, the LAD and LCX arise from the proximal part of the RCA. The last part of the modified Lipton classification identifies the course the vessel takes to reach its territory in relation to the great vessels (A: anterior, P: posterior, B: between, S: septal and C: combined) [1,12].

Our patient's anomaly does not fit into any of the above classifications as the RCA arises from the LAD. It then appears to course anteriorly across the right ventricular outflow tract (RVOT) to reach the atrioventricular (AV) groove as seen in Figures 1-3.

Although cardiac catheterization readily identifies these anomalies, coronary CT angiography is an important diagnostic tool to identify the specific course of this anomaly which would further guide management. Prognosis of this anomaly is dependent upon the course of the artery. If the RCA courses between the aorta and the pulmonary artery, the external compression might result in decreased coronary perfusion with resulting ischemia [6]. Another hypothesized cause of symptomatic ischemia is the acute angle of the RCA when turning towards the AV groove, resulting in reduced flow velocity and subsequent ischemic damage [10]. There was no evidence of significant stenosis in the RCA in our patient, and based on coronary angiography, it appeared to be coursing anteriorly in a benign fashion. Therefore, we elected to manage the patient conservatively with plans to obtain coronary CT angiography on an outpatient basis.

## Conclusions

In this report, we present a case of a patient who was found to have an anomalous RCA that branched off of the LAD. This is an extremely rare presentation and is more often than not benign in nature unless it takes an interarterial course between prominent vasculature. Coronary CT should be considered to identify the course of the anomalous artery and help guide management.
